# ICNP® nursing diagnoses and outcomes for community mental health based on the Tidal Model

**DOI:** 10.1590/0034-7167-2024-0519

**Published:** 2025-08-08

**Authors:** Carlon Washington Pinheiro, Ana Ruth Macedo Monteiro, Maria Célia de Freitas, Lúcia de Fátima da Silva, Nuno Damácio de Carvalho Félix, Rodrigo Jácob Moreira de Freitas

**Affiliations:** I Universidade Estadual do Ceará. Fortaleza, Ceará, Brazil; II Universidade Federal do Recôncavo da Bahia. Santo Antônio de Jesus, Bahia, Brazil; III Universidade do Estado do Rio Grande do Norte. Mossoró, Rio Grande do Norte, Brazil

**Keywords:** Psychiatric Nursing, Nursing Theory, Nursing Process, Nursing Diagnosis, Standardized Nursing Terminology., Enfermería Psiquiátrica, Teoría de Enfermería, Proceso de Enfermería, Diagnóstico de Enfermería, Terminología Normalizada de Enfermería.

## Abstract

**Objectives:**

to construct International Classification for Nursing Practice (ICNP®) nursing diagnosis and outcome statements for the context of community mental health based on the Tidal Model.

**Methods:**

methodological research, developed in three stages: identification of terms relevant to clinical nursing practice in the context of community mental health; mapping of selected terms with the terms of ICNP® 2019/2020; construction of nursing diagnosis and outcome statements.

**Results:**

1,417 terms were identified, resulting in a final number of 890 after the cross-mapping stage with ICNP® 2019/2020. A total of 426 nursing diagnosis/outcome statements and their respective definitions were constructed, categorizing them based on three concepts from the Tidal Model: self-domain, other-domain, and world-domain.

**Conclusions:**

the nursing diagnosis/outcome statements constructed reflect important concepts/terms of nursing practice in community mental health, contributing to the Nursing Process.

## RESUMEN

**Objetivos:**

construir enunciados de diagnósticos y resultados de enfermería de la Clasificación Internacional para la Práctica de Enfermería (CIPE®) para el contexto de salud mental comunitaria a partir del Tidal Model.

**Métodos:**

investigación metodológica, desarrollada en tres etapas: identificación de términos relevantes para la práctica clínica de enfermería en el contexto de la salud mental comunitaria; mapeo de términos seleccionados con términos de la CIPE® 2019/2020; construcción de diagnósticos de enfermería y declaraciones de resultados.

**Resultados:**

se identificaron 1.417 términos, resultando 890 luego de la etapa de mapeo cruzado con la CIPE® 2019/2020. Se construyeron 426 enunciados de diagnósticos/resultados de enfermería y sus respectivas definiciones, categorizándolos basado en tres conceptos del Tidal Model: dominio de uno mismo, dominio de los demás y dominio del mundo.

**Conclusiones:**

los diagnósticos/enunciados de resultados de enfermería construidos reflejan conceptos/términos importantes de la práctica de enfermería en salud mental comunitaria, contribuyendo al Proceso de Enfermería.

## INTRODUCTION

The International Classification for Nursing Practice (ICNP®) is defined as an enumerative and combinatorial terminology, representing a unifying framework for classification systems and elements of nursing practice, as it comprises Nursing Process (NP) elements through nursing diagnoses, outcomes and interventions. It is a classification recognized by the World Health Organization (WHO) and owned by the International Council of Nurses (ICN)^(1)^.

Since 2020, the ICN has entered into a partnership with the Systematized Nomenclature of Medicine International (SNOMED International), resulting in the integration of ICNP® terminology with SNOMED Clinical Terms (SNOMED CT), which became responsible for management, production and dissemination. However, the ICN remains responsible for maintaining control over the ICNP® content^(2)^.

Considering the context of the mental health specialty, it is clear that the development of nursing diagnoses (ND) and nursing outcomes (NO) of ICNP® can be advantageous, given their potential to represent psychosocial phenomena within NP, in addition to the possibility of being based on nursing theories. These aspects are in line with Resolutions 736/2024 and 678/2021 of the Federal Council of Nursing (In Portuguese, *Conselho Federal de Enfermagem* - COFEN)^(1,3-5)^.

Concerning the ICNP® subsets developed for mental health, the research^(3)^ that developed a subset for the context of alcoholics and the study^(6)^ that developed a subset focused on the care of people with mental disorders stand out. Some gaps stand out, such as the lack of foundation with nursing theories specific to the context of mental health, the absence of use of software in the stage of identifying concepts/terms and the lack of a database of concepts/terms with operational definitions for the specialty of community mental health.

Community mental health is understood as a field of knowledge that cannot be reduced to mental disorders or mental distress, expanding to the possibilities of promotion, protection, recovery and psychosocial rehabilitation. It highlights the value of the plurality of knowledge arising from different disciplines of knowledge and popular knowledge, in addition to the essential nature of community-based, territorialized services, with interprofessional, interdisciplinary and intersectoral assistance, being integrated into a Psychosocial Care Network (In Portuguese, *Rede de Atenção Psicossocial* - RAPS)^(7,8)^.

Considering the above, the Tidal Model theoretical framework was adopted, as it provides support for understanding phenomena in the context of mental health nursing, focusing on a person-centered view. The theory provides propositions that favor humanization, community care, social inclusion and overcoming the biomedical paradigm. These aspects demonstrate the synergy between the aforementioned theoretical framework and the context of public mental health policies in Brazil, especially considering the values of the Psychiatric Reform and the Anti-Asylum Struggle^(5,7,8)^.

## OBJECTIVES

To construct ICNP® ND/NO statements for the context of community mental health based on the Tidal Model.

## METHODS

### Ethical aspects

The study is subject to the prerogatives of national and international ethical guidelines, and the use of an Informed Consent Form is not applicable, as it did not involve human beings. Since this study is part of a larger research project, it was approved by the *Universidade Estadual do Ceará* Research Ethics Committee.

### Theoretical-methodological framework

The Tidal Model began to be developed by author Phil Barker between 1995 and 1997, and the first scientific studies were applied between 1997 and 1999, in psychiatric emergency services in England. It is a theoretical framework that explores the relationship between human experience and the ocean, considering that both are endowed with a natural flow, in constant movement and change. This being in the world of the ocean of human experiences brings the navigator the development of their being, in the face of their journey to become^(9)^.

It is characterized as a mid-range nursing theory, contextualized in the context of mental health, with application in projects in several countries around the world, such as Australia, Canada, New Zealand, Japan, Scotland and Wales. Among the main concepts, we can highlight the self-domain (personal and singular dimension), other-domain (social and interpersonal dimension) and world-domain (dimension of experience, of the interrelationship with the individual, people and environment), in addition to having theoretical propositions capable of directing its application in the context of mental health^(5,8)^.

### Study design and period

This is a methodological study of a descriptive nature, with an interface in the Brazilian method of developing ICNP® terminological subsets, carried out from August 2022 to February 2023. Three stages were completed: 1) identification of terms relevant to clinical nursing practice in the context of community mental health; 2) mapping of selected terms with terms of ICNP® 2019/2020; 3) construction of ND/NO statements^(10)^.

### Methodological procedures

The first stage involves identifying terms relevant to clinical nursing practice in the context of community mental health. The terms were collected through three empirical bases: literature, official documents and the ICNP® itself. Considering the empirical basis of literature, an integrative review was developed on standardized terminologies in nursing in the context of mental health. Based on the analysis of articles included in the integrative review, it became possible to select the main official documents used by studies to characterize public mental health policies in Brazil.

The following official documents were adopted: an ordinance from the Ministry of Health that addresses RAPS; the Ministry of Health’s basic care notebook specifically for mental health; two COFEN resolutions (one aimed at nursing specialties and another specifically on nursing team performance in mental health); and an article involving the Tidal Model theory (considering the need to represent terms/concepts of the theoretical framework).

The PorOnto® tool was used to extract and decompose terms from official documents. This tool performs the ontology construction process in eight phases. Phase 1 starts from the constructed *corpus* and in it specific filters can be used to process this *corpus* (it is worth noting that no filters were used in the *corpus* of this research). In phase 2, simple terms are extracted. In phase 3, compound terms are extracted (based on morphological sequence rules)^(11)^.

During phase 4, synonyms are selected, and in phase 5, terms corresponding to the Health Sciences Descriptors (DeCS) are selected. In phase 6, the general list of simple and compound terms is presented, along with synonyms, DeCS and other results^(11)^.

There was no construction of taxonomy based on compound terms, as occurs in phase 7 (this is an optional phase). Phase 8 is characterized by export in XLS or OWL format, with the XLS format being adopted. Subsequently, the terms were organized based on the ICNP® Seven-Axis Model, this process being carried out by the main researcher and later validated by another author of the study through consensus^(11)^.

We also manually identified terms in books, dissertations, theses and articles that already presented the use of ICNP® in some context that could be related to community mental health. The manual identification process occurred due to the incompatibility of some references for processing in PorOnto®, and this process was carried out by the main author and subsequently validated by the other researchers. Details of all references are available in the supplementary material.

The quantity of extracted terms was later entered into an electronic spreadsheet to organize, standardize, and standardize them. This process includes analyzing and excluding synonyms, adjusting verb tenses, grammatical genders, singular and plural, acronyms, expressions, or terms that belong exclusively to other categories of health professionals. This process began with the main author and was later checked by another author of the study, resulting in a consensus on the organization, standardization, and standardization process.

The second stage involves cross-mapping of terms, using two spreadsheets in Excel for Windows®: one with the terms extracted from the empirical bases adopted and another with the total list of terms from the ICNP® 2019/2020 Seven-Axis Model. Subsequently, they were imported into Access® 2013, which enabled the division between the extracted terms that were constant and non-constant in ICNP® 2019/2020.

The analysis was carried out using the equivalence degree assessment scale according to the International Organization for Standardization (ISO) 12.300/2016. One of the authors performed cross-mapping manually and assessed the degree of equivalence individually between the source terms and target terms, which were classified according to the respective degrees of equivalence^(12)^.

Degree 1 means total equivalence (lexical and conceptual) between the source term and the target term, and is attributed to extracted terms contained in ICNP® 2019/2020. Degree 2 was characterized as terms with equivalence in meaning, but with synonymy between the source term and the target term. Degree 3 was attributed when the source term was broader and had less specific meaning than the target term. Degree 4 was determined when the source term was more restricted and had more specific meaning than the target term. Degree 5 was analyzed as those terms in which no equivalence was found between the target term and the source term^(12)^.

Considering the high volume of terms analyzed, it was decided that another author would check the cross-mapping and classification of equivalence degrees. Finally, the constant and non-constant terms in ICNP® 2019/2020 were organized in alphabetical order, consolidating them into a term database. The first two stages were carried out between August and November 2022.

The development of the third stage involves the use of the term database to construct ND/NO statements, respecting ICN guidelines and ISO 18.104/2016 standards. It was decided to construct each statement with a positive and negative status to be used as both ND and NO, according to the relevance^(13)^.

The stage of constructing ND/NO statements continued with the performance of a new cross-mapping between the constructed and non-constant ND/NO statements in relation to pre-coordinated concepts of ICNP® 2019/2020. This was followed by the analysis based on the degree of equivalence assessment scale according to ISO 12.300/2016, resulting in a final list of constant and non-constant statements of ICNP® 2019/2020. This process was carried out manually by the main author, considering his experience in the context of community mental health, and reviewed by the other authors of the research through consensus^(12)^.

Operational definitions were constructed according to the stages adopted by the authors^(14)^, expressed in the form of theoretical statements. The following stages were adopted: 1) development of a preliminary definition; 2) literature review; 3) development or identification of specific characteristics; 4) mapping of concept meaning; and 5) affirmation of operational definition. The definitions were initially developed by the main researcher and subsequently checked and adapted by the other authors. The period of completion of the third stage occurred between December 2022 and February 2023.

### Data organization and analysis

The results were analyzed descriptively regarding the absolute and relative frequency of concepts, organized in charts and categorized according to three concepts of the Tidal Model (self-domain, other-domain and world-domain). Pre-coordinated ND/NO statements contained in ICNP® 2019/2020 will be represented followed by their respective classification codes expressed in terminology^(9,10)^.

## RESULTS

The term identification phase using PorOnto® and manual provided an initial quantity of 11,545 terms. After organizing, normalizing, standardizing and excluding terms that were not related to the context of community mental health, 1,417 terms were obtained.

During the cross-mapping stage, 303 constant terms and 1,114 non-constant terms were found in ICNP® 2019/2020. After analyzing the degree of equivalence in the cross-mapping process, the total number of terms was reduced to 890. It was found that, after the analysis, there are 520 (58.4%) terms that have some degree of equivalence (2, 3 and 4) in relation to ICNP® 2019/2020 terminology, in relation to the 370 (41.6%) that did not have any possible equivalence (analyzed as degree 5).

After obtaining the term database, the construction of 616 ND/NO statements began, carrying out a new cross-mapping between the statements constructed with pre-coordinated ND/NO of ICNP® 2019/2020, in which 176 (28.6%) statements were constant in the terminology and 440 (71.4%) were not constant.

The analysis was carried out according to the application of equivalence degree assessment scale in the cross-mapping process, resulting in a final quantity of 426 ND/NO statements, of which 146 (34.3%) have some degree of equivalence (2, 3 and 4) of pre-coordinated ND/NO statements in terminology and 280 (65.7%) do not have any possible equivalence (degree 5). The presentation of justifications for adopting the source ND/NO statements instead of ICNP® 2019/2020 target statements are present in supplementary material.

For each ND, a corresponding NO was constructed, and the framing of statements occurred based on the concepts of self-domain, other-domain and world-domain, coming from the Tidal Model^(9)^ theoretical framework, as represented in Chart 1.

**Chart 1:** Distribution of nursing diagnoses and nursing outcomes for community mental health based on the Tidal Model concepts, Fortaleza, Ceará, Brazil, 2024

Tidal Model concepts	Nursing diagnoses included in ICNP® 2019/2020
Self-domain	Drug Abuse (10022425); Impaired Acceptance of Health Status (10029480); Impaired Adaptation (10022027); Agitation (10025705); Allergy (10029697); Hallucination (10022500); Ambivalence (10047209); Spiritual Distress (10001652); Anxiety (10000477); Death Anxiety (10041017); Impaired Attention (10051501); Impaired Psychomotor Activity (10025087); Negative Self Image (10022724); Self Mutilation (10001623); Low Self Esteem (10029507); Low Self Control (10027469); Disrupted Energy Field (10001149); Impaired Ability to Manage Medication Regime (10022635); Impaired Ability to Participate in Care Planning (10035134); Impaired Protective Ability (10001014); Aggressive Behaviour (10047087); Self Destructive Behaviour (10027424); Compulsive Behaviour (10047189); Impaired Communication (10023370); Impaired Spiritual Status (10023336); Decisional Conflict (10000579); Confusion (10023633); Constipation (10000567); Impaired Impulse Control (10051538); Seizure (10045668); Conflicting Cultural Belief (10022397); Conflicting Health Belief (10022516); Conflicting Religious Belief (10021757); Sensory Deficit (10022730); Delirium (10022091); Impaired Dentition (10001131); Drug Dependence (10041381); Helplessness (10039952); Impaired Role Performance (10000941); Impaired Sexual Functioning (10001288); Impaired Adolescent Development (10023304); Impaired Human Development (10023260); Impaired Child Development (10023294); Hopelessness (10000742); Dehydration (10041882); Diarrhoea (10000630); Difficulty Coping (10001120); Pain (10023130); Medication Side Effect (10022626); Stigma (10022782); Fatigue (10000695); Lack of Appetite (10033399); Lack of Trust (10025947); Lack of Privacy (10025601); Hyperthermia (10000757); Depressed Mood (10022402); Delusion (10047002); Powerlessness (10001578); Excessive Food Intake (10000682); Deficient Food Intake (10000607); Impaired Fluid Intake (10029873); Adverse Medication Interaction (10042728); Activity Intolerance (10000431); Social Isolation (10001647); Impaired Health Maintenance (10000918); Fear (10000703); Impaired Memory (10001203); Nausea (10000859); Denial (10000624); Nightmare (10039968); Relationship Problem (10035744); Anger (10045578); Risk for Self Mutilation (10015318); Risk for Impaired School Performance (10041685); Risk for Impaired Child Development (10032317); Risk for Depressed Mood During Post Partum Period (10032338); Risk for Post Trauma Response (10015259); Risk to Be Victim of Neglect (10044452); Risk for Suicide (10015356); Risk for Violence (10022487); Altered Vital Sign (10050516); Stress Overload (10021742); Overweight (10027300); Impaired Socialisation (10001022); Impaired Sleep (10027226); Somnolence (10040141); Sadness (10040662); Shame (10046761); Vomiting (10025981).
Other-domain	Conflicting Family Attitude (10022456); Impaired Ability of Caregiver to Perform Caretaking (10035414); Impaired Community Coping (10034817); Impaired Family Coping (10034789); Caregiver Stress (10027773); Lack of Family Support (10022473); Impaired Parenting (10000939).
**Tidal Model concepts**	**Nursing diagnoses not included in ICNP® 2019/2020**
Self-domain	School Dropout; Drug Withdrawal; Akathisia; Impaired Medication Adherence; Impaired Medication Adherence; Hematological Change; Anxiety; Separation Anxiety; Impaired Learning; Ataxia; Impaired Autonomy; Impaired Self Realization; Bruxism; Bulimia; Impaired Ability to Perform Activities of Daily Living (specify); Impaired Ability to Participate; Catatonia; Aggressive Behaviour; Impaired Sexual Behaviour; Disorganized Behaviour; Repetitive Behaviour; Impaired Therapeutic Communication; Impaired Concentration; Vulnerable Social Condition; Impaired Knowledge of Sexual Education; Impaired Knowledge of the Asylum Model; Impaired Knowledge of Medications; Impaired Self Awareness; Impaired Growth; Impaired Creativity; Accidental Crisis; Maturational (Developmental) Crisis; Psychotic Crisis; Situational Crisis; Guilt; Impaired School Performance; Impaired Deinstitutionalization; Impaired Detoxification; Difficulty in Self Transcendence; Impaired Dignity in Death; Impaired Dignity; Dyskinesia; Discrimination; Edema; Impaired Elimination; Impaired Emancipation; Affective Blunting; Negative Emotion; Impaired Empathy; Manic State; Impaired Harm Reduction Strategy; Euphoria; Impaired Physical Exercise; Impaired Expression of Feeling; Lack of Problematization; Frustration; Hyperactivity; Hyperthyroidism; Hypoactivity; Hypochondriasis; Hypomania; Hypothyroidism; Oppressed Gender Identity; Impaired Personal Identity; Impaired Inclusion; Sexually Transmitted Infection; Drug Intoxication (Lithium Carbonate); Drug Intoxication; Impaired Language; Grief; Impaired Metabolism; Conflictual Migration; Neglect; Obesity; Obsession; Impaired Leisure Role; Impaired Thinking; Impaired Perception; Impaired Cardiac Process; Impaired Grief Process; Impaired Gastrointestinal System Process; Pruritus; Pseudocyesis (Pseudopregnancy); Psychosis; Impaired Reflection; Impaired Dietary Regime; Revictimization; Home Accident Risk; Accident Risk; Impaired Medication Regime Knowledge Risk; Risk for Impaired Adolescent Development; Risk for Medication Side Effects; Risk for Oppressed Gender Identity; Risk for Sexually Transmitted Infection; Risk for Adverse Medication Interaction; Risk for Drug Intoxication (Lithium Carbonate); Risk for Being a Victim of Sexual Assault (or Rape); Risk for Rights Violation; Impaired Routine; Impaired Marital Satisfaction; Loneliness; Somatization; Suicide Attempt; Rape Trauma; Impaired Bonding; Rights Violation; Impaired Volition.
Other-domain	Impaired Community Adaptation; Family Capacity to Participate in Care Planning; Impaired Family Communication; Impaired Caregiver Knowledge; Conflictual Coexistence; Impaired Co-Responsibility; Conflictual Family Belief; Family Crisis; Impaired Community Development; Impaired Caregiver Coping; Disrupted Popular Imagination; Impaired Community Leadership; Impaired Community Role; Impaired Group Role; Impaired Community Participation; Impaired Family Planning; Risk for Impaired Caregiver Knowledge; Risk for Impaired Community Development; Risk for Violence in the Community; Domestic Violence; Violence in the Community.
World-domain	Impaired Longitudinal Monitoring; Impaired Home Environment; Cultural Barriers; Ineffective Expanded Clinic; Impaired Ecology; Impaired Intersectoral Perspective; Impaired Psychosocial Rehabilitation.
**Tidal Model concepts**	**Nursing outcomes included in ICNP® 2019/2020**
Self-domain	No Drug Abuse (10028868); Acceptance of Health Status (10023499); Adherence to Medication Regime (10030192); Adherence to Therapeutic Regime (10030205); Reduced Agitation (10027843); No Allergy (10047492); Reduced Anxiety (10027858); Positive Appetite (10040333); Positive Self Esteem (10025751); Positive Self Image (10027108); No Self Mutilation (10029106); Effective Protective Ability (10028276); Able to Perform Health Maintenance (10023452); Effective Sexual Behaviour (10028187); Effective Spiritual Status (10028529); Effective Role Performance (10027940); Effective Human Development (10028572); Effective Child Development (10030222); No Diarrhoea (10040063); No Medication Side Effect (10040295); Effective Coping (10022378); Hope (10025780); Decreased Stress (10027929); No Fatigue (10034727); Adequate Hydration (10042065); Decreased Depressed Mood (10027901); Positive Personal Identity (10025664); Decreased Powerlessness (10027120); Improved Food Intake (10047324); Improved Fluid Intake (10047330); No Adverse Medication Interaction (10042991); Effective Memory (10028435); No Nausea (10028984); No Denial (10044260); Effective Sensory Perception (10028173); Improved Perception (10047437); Improved Response to Rape Trauma (10027668); Improved Response to Trauma (10027760); Adequate Sleep (10024930); Effective Activity Tolerance (10027634); Effective Decision Making (10028731); No Violence (10029168); No Vomiting (10029181).
Other-domain	Positive Family Support (10045702); Caregiver Able to Perform Caretaking (10035405); Effective Community Coping (10034801); Effective Caregiver Coping (10034838); Effective Family Coping (10034770); Reduced Caregiver Stress (10027794); Effective Parenting (10027955); Effective Family Process (10025232).
**Tidal Model concepts**	**Nursing outcomes not included in ICNP® 2019/2020**
Self-domain	Improved Akathisia; No Accident; Improved Adaptation; Improved Hematologic Change; Improved Hallucination; Decreased Distress; Improved Learning; Improved Ataxia; Improved Attention; Improved Psychomotor Activity; Effective Autonomy; Successful Self Actualization; Improved Bruxism; No Bulimia; Improved Energy Field; Ability to Perform Activities of Daily Living (specify) Successful; Ability to Manage Medication Regime Successful; Ability to Participate in Care Planning Successful; Ability to Participate Successful; Ability for Effective Protection; Improved Catatonia; Improved Aggressive Behaviour; Improved Self Destructive Behaviour; Improved Compulsive Behaviour; Improved Disorganized Behaviour; Improved Repetitive Behaviour; Effective Therapeutic Communication; Effective Communication; Effective Concentration; Improved Social Status; Improved Confidence; Improved Confusion; Improved Sex Education Knowledge; Improved Asylum Model Knowledge; Improved Medication Regime Knowledge; Increased Self Awareness; Improved Constipation; Effective Impulse Control; Effective Withdrawal Symptom Control; No Seizure; Positive Cultural Belief; Positive Health Belief; Positive Religious Belief; Effective Growth; Enhanced Creativity; Improved Accidental Crisis; Improved Maturation (Developmental) Crisis; Improved Psychotic Crisis; Improved Situational Crisis; Decreased Guilt; Improved Delusion; Improved Dentition; Improved Drug Dependence; Improved Helplessness; Effective School Performance; Improved Sexual Functioning; Effective Adolescent Development; Effective Deinstitutionalization; Effective Detoxification; Effective Elimination; Effective Emancipation; Improved Affective Blunting; Positive Emotion; Improved Empathy; Improved Manic State; Decreased Stigma; Effective Harm Reduction Strategy; Improved Euphoria; Improved Physical Exercise; Effective Expression of Feeling; Improved Frustration; Improved Hyperactivity; No Hyperthermia; No Hyperthyroidism; Improved Hypoactivity; Improved Hypochondriasis; Improved Hypomania; No Hypothyroidism; Strengthened Gender Identity; Effective Inclusion; No Sexually Transmitted Infection; No Drug Intoxication (Lithium Carbonate); No Drug Intoxication; Improved Language; Improved Grief; Decreased Fear; Improved Metabolism; Improved Migration; No Neglect; Improved Obsession; Effective Leisure Role; Improved Thinking; Decreased Nightmare; Effective Weight; Improved Privacy; Improved Problematization; Effective Cardiac Process; Improved Grief Process; Positive Gastrointestinal System Process; No Pruritus; Improved Pseudocyesis (Psychological Pregnancy); Improved Psychosis; Decreased Anger; Improved Reflection; Improved Dietary Regime; Positive Relationship; No Revictimization; Improved Routine; Improved Marital Satisfaction; Improved Vital Sign (Vital Signs); Effective Socialization; Decreased Loneliness; Improved Somatization; Improved Somnolence; No Suicide Attempt; No Rape Trauma; Improved Sadness; Enhanced Shame; Satisfactory Bond; No Rights Violation; No Rights Violation; Enhanced Volition.
Other-domain	Facilitated Community Adaptation; Improved Family Attitude; Family Capacity to Participate in Successful Care Planning; Effective Family Communication; Strengthened Caregiver Knowledge; Improved Coexistence; Effective Co-Responsibility; Positive Family Belief; Effective Community Development; Strengthened Popular Imagination; Effective Community Leadership; Effective Community Role; Effective Group Role; Improved Community Participation; Effective Family Planning; No Domestic Violence; Improved Community Violence.
World-domain	Improved Longitudinal Monitoring; Improved Home Environment; No Cultural Barriers; Effective Expanded Clinic; Improved Ecology; Effective Intersectoral Perspective; Successful Psychosocial Rehabilitation.

ND/NO statements, with their respective operational definitions and classifications with the Tidal Model, are fully displayed in the supplementary material section.

## DISCUSSION

The findings of this study show the majority of 520 (58.4%) terms/concepts of ICNP®, originating from the Seven-Axis Model, in relation to identified terms/concepts. The same did not happen in relation to pre-coordinated ICNP® statements, since only 146 (34.3%) of constructed ND/NO statements had some equivalence with pre-coordinated statements of terminology.

It is important to highlight that the reduced number of pre-coordinated ICNP® statements does not mean an absence of terminology, given the large number of terms from the focus axis that served as a basis for the construction of the new ND/NO statements. Examples are: Withdrawal (10035422); Separation Anxiety (10017880); Distress (10006118); Bulimia (10003759); Catatonia (10004056); Disorganised Behaviour (10006059); Concentration (10004910); School Performance (10017559); Discrimination (10006037); Hyperactivity (10009302); Hypoactivity (10009466)^(1)^.

Given the representation within the Seven-Axis Model, it is worth noting that the number of 440 (71.4%) non-constant ND/NO statements may indicate that the context of the community mental health specialty has important specificities that demand greater representation in pre-coordinated ICNP® statements^(6,7)^.

Among these specificities of the specialty context, it is possible to highlight the importance of creating the following ND/NO: Drug Poisoning (Lithium Carbonate); Risk for Drug Poisoning (Lithium Carbonate); No Drug Poisoning (Lithium Carbonate); Hematological Change; and Improved Hematological Change. These statements are related to the repercussions of some psychiatric medications that directly affect the health-disease process of users, in addition to being part of the routine of nurses who work in the area^(15)^.

ND/NO statements, such as Impaired Knowledge of Medication Regime, Impaired Risk for Knowledge of Medication Regime and Improved Knowledge of Medication Regime, reinforce the possibilities in the scope of psychoeducation, being another routine dimension of the practice of nurses who work in mental health^(5,15)^.

From the construction of ND/NO statements, such as Accidental Crisis, Maturational (Developmental) Crisis, Situational Crisis, Improved Accidental Crisis, Improved Maturational (Developmental) Crisis and Improved Situational Crisis, it became evident that these are examples of routine phenomena that are part of the vocabulary of the mental health context, but that had not yet been represented as ND/NO statements in ICNP® 2019/2020^(15)^.

The set of ND/NO related to the concept of self-mastery had a greater quantity, as it represents terms/concepts and clinical findings that concern the personal, private and individualized dimension of human beings, which is in a constant dynamic of reconstruction and change^(9)^. The set of ND/NO related to the other-domain focuses on representing terms/concepts that address interpersonal and social dynamics, covering aspects of social, family, professional, community roles, among others^(5,8)^.

World mastery is a concept based on the understanding of the world of experience in a broad way, permeating processes related to the mastery of the self and others, being the means of sharing the experiences lived by each person and collectives in a unique way^(5,9)^.

The framing of ND/NO statements in three concepts of the Tidal Model provides parsimony and flexibility for a less restrictive use, especially when considering the multiplicity of theoretical approaches in mental health. In relation to ND/NO statements constructed based on the Tidal Model epistemological foundations, the following stand out: Impaired Self Realization; Successful Self Realization; Difficulty in Self Transcendence; Successful Self Transcendence; Effective Emancipation; Impaired Emancipation^(1,10)^.

The construction of ND/NO statements for the context of mental health is relevant not only in relation to the clinical findings present in the specialty, but also in relation to paradigms existing in the Brazilian mental health policy: in the Brazilian Health System RAPS. These nuances can be perceived from the following ND/NO: Impaired Deinstitutionalization; Effective Deinstitutionalization; Impaired Co-responsibility; Effective Co-responsibility; Impaired Psychosocial Rehabilitation; Successful Psychosocial Rehabilitation; among others^(5,7)^.

The RAPS is composed of a set of levels of health care aimed at assisting people with mental health problems and problematic drug use. It includes basic care, psychosocial care, emergency care, temporary residential care, hospital care, deinstitutionalization strategies and psychosocial rehabilitation strategies. The construction of ND/NO statements sought to value the practice of the different services involved in the care network, including the different types of Psychosocial Care Centers^(5,7)^.

Considering the incorporation of ICNP® ND/NO into health information systems, their relevance in relation to the implementation of NP in clinical practice stands out, given that they are based on ISO standards. These standards provide standardization of diagnostic statements, outcomes and nursing actions according to a computational representation of nursing practice^(16)^.

This standardization favors retrievable and interoperable electronic documentation, in addition to standardization of terms/concepts of nursing practice. The use of a unified language promotes improved communication among professionals and between professional and patient, positively impacting patient safety, mitigating errors and risks of unnecessary harm associated with health care^(17)^.

It is understood that the ICNP® ND/NO also provide gains when exploring different concepts and phenomena of professional practice, and can become a conceptual basis for the creation of nursing theories, as seen in a study^(18)^ that describes a theorizing strategy that integrates components of classifications and terminologies with elements of large and medium-range nursing theories.

Given the above, it is clear that these different aspects addressed in relation to ND/NO statements for community mental health are in line with the WHO World Mental Health Report and its Comprehensive Action Plan for Mental Health (2013-2030), contemplating three proposed general objectives, namely: 1) to provide comprehensive, integrated and responsive mental health and social care in community contexts; 2) to implement strategies for promotion and prevention in mental health; and 3) to strengthen information, evidence and research systems for mental health^(19)^.

### Study limitations

One limitation is the need to align the results with the new ISO 18.104:2023, as it had not been published in time for the completion of this study. It is evident that the rigor of this study allows the alignment of results to address these limitations in the future structuring of the ICNP® terminology subset. The process of validating the content of operational definitions will be the subject of future research, given its importance for safety in clinical practice.

### Contributions to nursing

The construction of ICNP® ND/NO, associated with the Tidal Model theoretical framework, becomes a strategy that enhances NP in community mental health, and can favor the uniqueness of care, the organization of work processes, patient safety and the improvement of the quality of professional records, in addition to facilitating the dissemination of knowledge in the specialty.

## CONCLUSIONS

As intended, 426 ICNP® ND/NO statements were constructed for the context of community mental health, and categorized using the Tidal Model theory. ND/NO statements related to the self-domain had the highest number, followed by the other-domain and the world-domain. It was evident that most of pre-coordinated ICNP® ND/NO statements were not included in ICNP® 2019/2020 terminology. The research will continue in ND/NO content validity and in nursing intervention construction and validity.

## Data Availability

The research data are available in a repository: https://doi.org/10.48331/scielodata.NHZIL6.
