# ERRATA

**DOI:** 10.1590/0034-7167.20257805e08pt

**Published:** 2025-10-20

No artigo “Diagnósticos e resultados de enfermagem da CIPE^®^ para saúde mental comunitária fundamentada no Tidal Model”, com número DOI: https://doi.org/10.1590/0034-7167-2024-0519pt, publicado no periódico Revista Brasileira de Enfermagem, 2025;78(4):e20240519, somente na versão em português, na página 4, Quadro 1:

Onde se lia:

**Table:** 

**Conceitos do *Tidal Model***	**Resultados de enfermagem constantes na CIPE® 2019/2020**

Lê-se:

**Table:** 

**Conceitos do *Tidal Model***	**Diagnósticos de enfermagem constantes na CIPE® 2019/2020**

