# Supplementary material for: ICNP® nursing diagnoses and outcomes for community mental health based on the Tidal Model
Source: Rev Bras Enferm. 2025 Aug 8;78(4):e20240519. doi: 10.1590/0034-7167-2024-0519 (PMC12334136; doi:10.1590/0034-7167-2024-0519)
Supplement: Translation

# Translation: Diagnósticos e resultados de enfermagem da CIPE® para saúde mental comunitária fundamentada no *Tidal Model*

**DOI:** 10.1590/0034-7167-2024-0519pt

**Authors:** Carlon Washington Pinheiro, Ana Ruth Macedo Monteiro, Maria Célia de Freitas, Lúcia de Fátima da Silva, Nuno Damácio de Carvalho Félix, Rodrigo Jácob Moreira de Freitas

**Affiliations:** I Universidade Estadual do Ceará. Fortaleza, Ceará, Brasil; II Universidade Federal do Recôncavo da Bahia. Santo Antônio de Jesus, Bahia, Brasil; III Universidade do Estado do Rio Grande do Norte. Mossoró, Rio Grande do Norte, Brasil

**Keywords:** Enfermagem Psiquiátrica, Teoria de Enfermagem, Processo de Enfermagem, Diagnóstico de Enfermagem, Terminologia Padronizada em Enfermagem.

## Abstract

**Objetivos:**

construir enunciados de diagnósticos e resultados de enfermagem da Classificação Internacional para a Prática de Enfermagem (CIPE®) para o contexto da saúde mental comunitária fundamentado no *Tidal Model*.

**Métodos:**

pesquisa metodológica, desenvolvida em três etapas: identificação de termos relevantes para a prática clínica de enfermagem no contexto da saúde mental comunitária; mapeamento dos termos selecionados com os termos da CIPE® 2019/2020; construção dos enunciados de diagnósticos e resultados de enfermagem.

**Resultados:**

identificaram-se 1.417 termos, obtendo-se posteriormente um quantitativo final de 890 após a etapa de mapeamento cruzado com a CIPE® 2019/2020. Realizou-se a construção de 426 enunciados de diagnósticos/resultados de enfermagem e suas respectivas definições, categorizando-os a partir de três conceitos do *Tidal Model:* o domínio do eu, domínio dos outros e domínio do mundo.

**Conclusões:**

os enunciados diagnósticos/resultados de enfermagem construídos refletem conceitos/termos importantes da prática de enfermagem em saúde mental comunitária, contribuindo para o Processo de Enfermagem.

## INTRODUÇÃO

A Classificação Internacional para a Prática de Enfermagem (CIPE®) é definida como uma terminologia enumerativa e combinatória, representando um marco unificador dos sistemas de classificação e elementos da prática de enfermagem, pois compreende elementos do Processo de Enfermagem (PE) por meio de diagnósticos, resultados e intervenções de enfermagem. É uma classificação reconhecida pela Organização Mundial da Saúde (OMS), e sua propriedade é do Conselho Internacional de Enfermeiros (CIE)^(1)^.

O CIE, desde 2020, firmou uma parceria com a *Systematized Nomenclature of Medicine International* (SNOMED *International*), resultando na integração da terminologia CIPE® com a SNOMED *Clinical Terms* (SNOMED CT), que passou a ser responsável pelo gerenciamento, produção e divulgação. No entanto, coube ao CIE a responsabilidade de manter o controle do conteúdo da CIPE®^(2)^.

Considerando o contexto da especialidade de saúde mental, percebe-se que o desenvolvimento de diagnósticos de enfermagem (DE) e resultados de enfermagem (RE) da CIPE® pode ser vantajoso, tendo em vista sua potencialidade para representar os fenômenos psicossociais dentro do PE, além da possibilidade de embasamento em teorias de enfermagem. Esses aspectos estão em consonância com as Resoluções n^o^ 736/2024 e n^o^ 678/2021 do Conselho Federal de Enfermagem (COFEN)^(1,3-5)^.

Em relação aos subconjuntos CIPE® desenvolvidos para a saúde mental, evidenciam-se a pesquisa^(3)^, que desenvolveu um subconjunto para o contexto da pessoa alcoolista, e o estudo^(6)^, que desenvolveu um subconjunto voltado para o cuidado a pessoas portadoras de transtornos mentais. Destacam-se algumas lacunas, como falta de fundamentação com teorias de enfermagem específicas do contexto da saúde mental, ausência de utilização de *software* na etapa de identificação de conceitos/termos e falta de um banco de conceitos/termos com definições operacionais para a especialidade de saúde mental comunitária.

Entende-se saúde mental comunitária como um campo do conhecimento que não pode ser reduzido ao transtorno mental ou sofrimento mental, ampliando-se para as possibilidades de promoção, proteção, recuperação e reabilitação psicossocial. Destaca-se a valorização da pluralidade de saberes advindos de diferentes disciplinas do conhecimento e do saber popular, além da imprescindibilidade dos serviços de base comunitária, territorializados, com assistência interprofissional, interdisciplinar e intersetorial, sendo integrada a uma Rede de Atenção Psicossocial (RAPS)^(7,8)^.

Diante do exposto, adotou-se o referencial teórico *Tidal Model*, pois fornece subsídios para compreender os fenômenos no contexto da enfermagem em saúde mental, direcionando-se para uma visão centrada na pessoa. A teoria fornece proposições que favorecem a humanização, o cuidado comunitário, a inclusão social e a superação do paradigma biomédico. Tais aspectos evidenciam a sinergia entre o referencial teórico citado e o contexto das políticas públicas de saúde mental do Brasil, principalmente considerando os valores da Reforma Psiquiátrica e da Luta Antimanicomial^(5,7,8)^.

## OBJETIVOS

Construir enunciados de DE/RE da CIPE® para o contexto da saúde mental comunitária fundamentado no *Tidal Model*.

## MÉTODOS

### Aspectos éticos

O estudo está sujeito às prerrogativas das diretrizes éticas nacionais e internacionais, sendo não aplicável a utilização do Termo de Consentimento Livre e Esclarecido, pois não envolveu seres humanos. Em virtude de este estudo fazer parte de um projeto de pesquisa mais abrangente, houve a aprovação pelo Comitê de Ética em Pesquisa da Universidade Estadual do Ceará.

### Referencial teórico-metodológico

O *Tidal Model* começou a ser desenvolvido pelo autor Phil Barker entre os anos de 1995 e 1997, e os primeiros estudos científicos foram aplicados entre os anos de 1997 e 1999, em serviços de emergência psiquiátrica na Inglaterra. É um referencial teórico que explora a relação entre a experiência humana e o oceano, tendo em vista que ambas são dotadas de um fluxo natural, em constante movimento e mudança. Esse estar no mundo do oceano de experiências humanas traz ao navegante o desenvolvimento de seu ser, diante de sua jornada para tornar-se^(9)^.

Caracteriza-se como uma teoria de enfermagem de médio alcance, contextualizada no âmbito da saúde mental, com aplicação em projetos em diversos países do mundo, como Austrália, Canadá, Nova Zelândia, Japão, Escócia e País de Gales. Entre os principais conceitos, podem-se destacar o domínio do eu (dimensão pessoal e singular), o domínio dos outros (dimensão social e interpessoal) e o domínio do mundo (dimensão da experiência, da inter-relação com o indivíduo, pessoas e ambiente), além de contar com proposições teóricas capazes de direcionar sua aplicação no contexto da saúde mental^(5,8)^.

### Desenho do estudo e período

Estudo metodológico de caráter descritivo, com interface no método brasileiro de desenvolvimento de subconjuntos terminológicos da CIPE®, realizado no período compreendendo agosto de 2022 até fevereiro de 2023. Cumpriram-se três etapas: 1) identificação de termos relevantes para a prática clínica de enfermagem no contexto da saúde mental comunitária; 2) mapeamento dos termos selecionados com os termos da CIPE® 2019/2020; 3) construção dos enunciados de DE/RE^(10)^.

### Procedimentos metodológicos

A primeira etapa compreende a identificação dos termos relevantes para a prática clínica de enfermagem no contexto da saúde mental comunitária. Os termos foram coletados por meio de três bases empíricas: a literatura, documentos oficiais e a própria CIPE®. Considerando a base empírica da literatura, desenvolveu-se revisão integrativa sobre a temática de terminologias padronizadas em enfermagem no contexto da saúde mental. A partir da análise dos artigos incluídos na revisão integrativa, tornou-se possível selecionar os principais documentos oficiais utilizados pelos estudos para caracterizar as políticas públicas de saúde mental no Brasil.

Adotaram-se os seguintes documentos oficiais: uma portaria do Ministério da Saúde que aborda a RAPS; o caderno da atenção básica do Ministério da Saúde específico para saúde mental; duas resoluções do COFEN (uma destinada às especialidades de enfermagem e outra específica sobre a atuação da equipe de enfermagem em saúde mental); e um artigo envolvendo a teoria do *Tidal Model* (considerando a necessidade de representação de termos/conceitos do referencial teórico).

Utilizou-se a ferramenta PorOnto® para extrair e decompor termos dos documentos oficiais. Essa ferramenta realiza o processo de construção da ontologia em oito fases. A fase 1 parte do *corpus* construído e nela podem ser utilizados filtros específicos para processar esse *corpus* (destaca-se que não houve utilização de filtros no *corpus* desta pesquisa). Na fase 2, ocorre a extração de termos simples. Na fase 3, ocorre a extração dos termos compostos (baseados nas regras de sequência morfológica)^(11)^.

Durante a fase 4, ocorre a seleção de sinônimos, e na fase 5, a seleção de termos correspondentes com os Descritores em Ciências da Saúde (DeCS). Na fase 6, a lista geral de termos simples e composta é apresentada, juntamente com os sinônimos, os DeCS e entre outros resultados^(11)^.

Não houve a construção da taxonomia baseada nos termos compostos, conforme ocorre na fase 7 (trata-se de fase opcional). A fase 8 é caracterizada pela exportação em formato XLS ou OWL, sendo adotado o formato XLS. Posteriormente, os termos foram organizados a partir do Modelo de Sete Eixos da CIPE®, sendo esse processo realizado pelo pesquisador principal e posteriormente validado por outro autor do estudo por meio de consenso^(11)^.

Realizou-se também a identificação manual de termos em livros, dissertações, tese e artigos que já apresentavam o uso da CIPE® em algum contexto que pudesse ser relacionado à saúde mental comunitária. O processo de identificação manual ocorreu por conta da inviabilidade de compatibilidade de algumas referências para processamento no PorOnto®, sendo esse processo realizado pelo autor principal e validado posteriormente pelos demais pesquisadores. O detalhamento de todas as referências está disponibilizado no material suplementar.

O quantitativo de termos extraídos foi posteriormente colocado em uma planilha eletrônica para realizar a organização, normalização e uniformização. Esse processo inclui a análise e exclusão de sinônimos, adequação dos tempos verbais, dos gêneros gramaticais, de singular e plural, de siglas, de expressões ou termos que pertencem exclusivamente a outras categorias de profissionais de saúde. Esse processo se iniciou com o autor principal, sendo posteriormente checado por outro autor do estudo, havendo um consenso sobre o processo de organização, normalização e uniformização.

A segunda etapa contempla o mapeamento cruzado dos termos, sendo utilizadas duas planilhas no programa *Excel for Windows*®: uma com os termos extraídos das bases empíricas adotadas e outra com a lista total de termos do Modelo de Sete Eixos da CIPE® 2019/2020. Posteriormente, foram importadas para o programa *Access*® 2013, que viabilizou a divisão entre os termos extraídos que eram constantes e não constantes da CIPE® 2019/2020.

Seguiu-se para a análise utilizando a escala de avaliação de grau de equivalência segundo a Norma *International Organization for Standardization* (ISO) 12.300/2016. Um dos autores realizou o mapeamento cruzado manualmente e avaliou o grau de equivalência individualmente entre os termos fonte e termos-alvo, sendo classificados nos respectivos graus de equivalência^(12)^.

O grau 1 significa equivalência total (léxica e conceitual) entre o termo fonte e o termo-alvo, sendo atribuídos aos termos extraídos constantes na CIPE® 2019/2020. Caracterizaram-se como grau 2 os termos com equivalência de significado, mas com sinonímia entre o termo fonte e o termo-alvo. Atribuiu-se grau 3 quando o termo fonte era mais amplo e tinha menos significado específico do que o termo-alvo. Determinou-se grau 4 quando o termo fonte era mais restrito e tinha mais significado específico do que o termo-alvo. Analisaram-se como grau 5 aqueles termos em que nenhuma equivalência foi encontrada entre o termo-alvo e o termo fonte^(12)^.

Considerando o alto volume de termos analisados, estabeleceu-se que um outro autor fizesse a checagem do mapeamento cruzado e da classificação dos graus de equivalência. Por fim, os termos constantes e não constantes na CIPE® 2019/2020 foram organizadas em ordem alfabética, consolidando-se em banco de termos. O período de realização das duas primeiras etapas ocorreu entre agosto e novembro de 2022.

O desenvolvimento da terceira etapa engloba a utilização do banco de termos para construção de enunciados de DE/RE, respeitando as diretrizes da CIE e as normas da ISO 18.104/2016. Optou-se por construir cada enunciado com um status positivo e negativo para serem utilizados tanto como DE como RE, de acordo com a pertinência^(13)^.

A etapa de construção dos enunciados de DE/RE prosseguiu com a realização de um novo mapeamento cruzado entre os enunciados de DE/RE construídos e não constantes em relação aos conceitos pré-coordenados da CIPE® 2019/2020. Seguiu-se com a análise a partir da escala de avaliação de grau de equivalência segundo a ISO 12.300/2016, resultando em uma lista final de enunciados constantes e não constantes da CIPE® 2019/2020. Esse processo foi realizado pelo autor principal de forma manual, considerando sua experiência no contexto da saúde mental comunitária, e revisado pelos demais autores da pesquisa por meio de consenso^(12)^.

As definições operacionais foram construídas segundo as etapas adotadas pelos autores^(14)^, expressas na forma de afirmações teóricas. Adotaram-se as seguintes etapas: 1) desenvolvimento de uma definição preliminar; 2) revisão da literatura; 3) desenvolvimento ou identificação de características específicas; 4) mapeamento do significado do conceito; e 5) afirmação da definição operacional. As definições foram elaboradas, inicialmente, pelo pesquisador principal, e, posteriormente, checadas e adaptadas pelos demais autores. O período de realização da terceira etapa ocorreu entre dezembro de 2022 e fevereiro de 2023.

### Organização e análise de dados

Os resultados foram analisados descritivamente quanto à frequência absoluta e relativa dos conceitos, organizados em quadros e categorizados segundo três conceitos do *Tidal Model* (domínio do eu, domínio dos outros e domínio do mundo). Os enunciados de DE/RE pré-coordenados constantes na CIPE® 2019/2020 serão representados seguidos com seus devidos códigos de classificação expressos na terminologia^(9,10)^.

## RESULTADOS

A fase de identificação de termos pelo *software* PorOnto® e manual proporcionou um quantitativo inicial de 11.545 termos. Após realizar a organização, normalização, uniformização e exclusão de termos que não fossem relacionados ao contexto da saúde mental comunitária, resultou-se uma quantidade de 1.417 termos.

Durante a etapa de mapeamento cruzado, encontraram-se 303 termos constantes e 1.114 não constantes na CIPE® 2019/2020. Após a análise do grau de equivalência no processo de mapeamento cruzado, reduziu-se o número total de termos para 890. Constatou-se que, após a análise, existem 520 (58,4%) termos que possuem algum grau de equivalência (2,3 e 4) em relação à terminologia CIPE® 2019/2020, em relação aos 370 (41,6%) que não possuíram nenhuma equivalência possível (analisados como grau 5).

A partir da obtenção do banco de termos, iniciou-se a construção de 616 enunciados de DE/RE, procedendo-se a um novo mapeamento cruzado entre os enunciados construídos com os DE/RE pré-coordenados da CIPE® 2019/2020, em que 176 (28,6%) dos enunciados eram constantes na terminologia e 440 (71,4%) não eram constantes.

Seguiu-se para a análise segundo a aplicação da escala de avaliação de grau de equivalência no processo de mapeamento cruzado, resultando na obtenção de um quantitativo final de 426 de enunciados de DE/RE, sendo que 146 (34,3%) possuem algum grau de equivalência (2, 3 e 4) de enunciados de DE/RE pré-coordenados na terminologia e 280 (65,7%) não possuem nenhuma equivalência possível (grau 5). A apresentação das justificativas sobre adoção dos enunciados de DE/RE fonte em vez dos enunciados alvo CIPE® 2019/2020 estão presentes no material suplementar.

Para cada DE, houve a construção de um RE correspondente, e o enquadramento dos enunciados ocorreu a partir dos conceitos do domínio do eu, domínio dos outros e domínio do mundo, provenientes do referencial teórico *Tidal Model* ^(9)^, conforme representado no Quadro 1.

**Quadro 1:** Distribuição dos diagnósticos de enfermagem e resultados de enfermagem para saúde mental comunitária baseado nos conceitos do *Tidal Model*, Fortaleza, Ceará, Brasil, 2024

Conceitos do *Tidal Model*	Resultados de enfermagem constantes na CIPE® 2019/2020
Domínio do eu	Abuso de Drogas (10022425); Aceitação da Condição de Saúde, Prejudicada (10029480); Adaptação, Prejudicada (10022027); Agitação (10025705); Alergia (10029697); Alucinação (10022500); Ambivalência (10047209); Angústia Espiritual (10001652); Ansiedade (10000477); Ansiedade Face à Morte (10041017); Atenção, Prejudicada (10051501); Atividade Psicomotora, Prejudicada (10025087); Autoimagem, Negativa (10022724); Automutilação (10001623); Baixa Autoestima (10029507); Baixo Autocontrole (10027469); Campo de Energia, Interrompido (10001149); Capacidade para Manejar (Controlar) o Regime Medicamentoso, Prejudicada (10022635); Capacidade para Participar no Planejamento do Cuidado, Prejudicada (10035134); Capacidade para Proteção, Prejudicada (10001014); Comportamento, Agressivo (10047087) Comportamento, Autodestrutivo (10027424); Comportamento, Compulsivo (10047189); Comunicação, Prejudicada (10023370); Condição Espiritual, Prejudicada (10023336); Conflito de Decisão (10000579); Confusão (10023633); Constipação (10000567); Controle do Impulso, Prejudicado (10051538); Convulsão (10045668); Crença Cultural, Conflituosa (10022397); Crença de Saúde, Conflituosa (10022516); Crença Religiosa, Conflituosa (10021757); Déficit Sensorial (10022730); Delírio (10022091); Dentição, Prejudicada (10001131); Dependência de Drogas (10041381); Desamparo (10039952); Desempenho de Papel, Prejudicado (10000941); Desempenho Sexual, Prejudicado (10001288); Desenvolvimento do Adolescente, Prejudicado (10023304); Desenvolvimento Humano, Prejudicado (10023260); Desenvolvimento Infantil, Prejudicado (10023294); Desesperança (10000742); Desidratação (10041882); Diarreia (10000630); Dificuldade de Enfrentamento (10001120); Dor (10023130); Efeito Colateral da Medicação (10022626); Estigma (10022782); Fadiga (10000695); Falta de Apetite (10033399); Falta de Confiança (10025947); Falta de Privacidade (10025601); Hipertermia (10000757); Humor, Deprimido (10022402); Ilusão (10047002); Impotência (10001578); Ingestão de Alimentos, Excessiva (10000682); Ingestão de Alimentos, Insuficiente (ou Deficitária) (10000607); Ingestão de Líquidos, Prejudicada (10029873); Interação Medicamentosa, Adversa (10042728); Intolerância à Atividade (10000431); Isolamento Social (10001647); Manutenção da Saúde, Prejudicada (10000918); Medo (10000703); Memória, Prejudicada (10001203); Náusea (10000859); Negação (10000624); Pesadelo (10039968); Problema de Relacionamento (10035744); Raiva (10045578); Risco de Automutilação (10015318); Risco de Desempenho Escolar, Prejudicado (10041685); Risco de Desenvolvimento Infantil, Prejudicado (10032317); Risco de Humor Deprimido, no Período Pós-Parto (10032338); Risco de Resposta Pós-Trauma (10015259); Risco de ser Vítima de Negligência (10044452); Risco de Suicídio (10015356); Risco de Violência (10022487); Sinal Vital (Sinais Vitais), Alterado (10050516); Sobrecarga de Estresse (10021742); Sobrepeso (10027300); Socialização, Prejudicada (10001022); Sono, Prejudicado (10027226); Sonolência (10040141); Tristeza (10040662); Vergonha (10046761); Vômito (10025981).
Domínio dos outros	Atitude Familiar, Conflituosa (10022456); Capacidade do Cuidador para Executar o Cuidado, Prejudicada (10035414); Enfrentamento Comunitário, Prejudicado (10034817); Enfrentamento Familiar, Prejudicado (10034789); Estresse do Cuidador (10027773); Falta de Apoio Familiar (10022473); Parentalidade, Prejudicada (10000939).
**Conceitos do *Tidal Model***	**Diagnósticos de enfermagem não constantes na CIPE® 2019/2020**
Domínio do eu	Abandono escolar; Abstinência de Drogas; Acatisia; Adesão ao Regime Medicamentoso, Prejudicada; Adesão ao Regime Terapêutico, Prejudicada; Alteração Hematológica; Angústia; Ansiedade de Separação; Aprendizagem, Prejudicada; Ataxia; Autonomia, Prejudicada; Autorrealização, Prejudicada; Bruxismo; Bulimia; Capacidade para Executar Atividades de Vida Diária (especificar), Prejudicada; Capacidade para Participar, Prejudicada; Catatonia; Comportamento Agressivo; Comportamento Sexual, Prejudicado; Comportamento, Desorganizado; Comportamento, Repetitivo; Comunicação Terapêutica, Prejudicada; Concentração, Prejudicada; Condição Social, Vulnerável; Conhecimento sobre Educação Sexual, Prejudicado; Conhecimento sobre Modelo Manicomial, Prejudicado; Conhecimento sobre Regime Medicamentoso, Prejudicado; Consciência de Si Mesmo, Prejudicada; Crescimento, Prejudicado; Criatividade, Prejudicada; Crise Acidental; Crise Maturacional (de Desenvolvimento); Crise Psicótica; Crise Situacional; Culpa; Desempenho Escolar, Prejudicado; Desinstitucionalização, Prejudicada; Desintoxicação, Prejudicada; Dificuldade na Autrotranscendência; Dignidade na Morte, Prejudicada; Dignidade, Prejudicada; Discinesia; Discriminação; Edema; Eliminação, Prejudicada; Emancipação, Prejudicada; Embotamento Afetivo; Emoção, Negativa; Empatia, Prejudicada; Estado Maníaco; Estratégia de Redução de Danos, Prejudicada; Euforia; Exercício Físico, Prejudicado; Expressão de Sentimento, Prejudicada; Falta de Problematização; Frustração; Hiperatividade; Hipertireoidismo; Hipoatividade; Hipocondria; Hipomania; Hipotireoidismo; Identidade de Gênero, Oprimida; Identidade Pessoal, Prejudicada; Inclusão, Prejudicada; Infecção Sexualmente Transmissível; Intoxicação Medicamentosa (Carbonato de Lítio); Intoxicação Medicamentosa; Linguagem, Prejudicada; Mágoa; Metabolismo, Prejudicado; Migração, Conflituosa; Negligência; Obesidade; Obsessão; Papel de Lazer, Prejudicado; Pensamento, Prejudicado; Percepção, Prejudicada; Processo Cardíaco, Prejudicado; Processo de Luto, Prejudicado; Processo do Sistema Gastrointestinal, Prejudicado; Prurido; Pseudociese (Gravidez Psicológica); Psicose; Reflexão, Prejudicada; Regime Dietético, Prejudicado; Revitimização; Risco de Acidente Doméstico; Risco de Acidente; Risco de Conhecimento sobre Regime Medicamentoso, Prejudicado; Risco de Desenvolvimento do Adolescente, Prejudicado; Risco de Efeito Colateral da Medicação; Risco de Identidade de Gênero, Oprimida; Risco de Infecção Sexualmente Transmissível; Risco de Interação Medicamentosa, Adversa; Risco de Intoxicação Medicamentosa (Carbonato de Lítio); Risco de ser vítima de agressão sexual (ou estupro); Risco de Violação de Direitos; Rotina, Prejudicada; Satisfação Conjugal, Prejudicada; Solidão; Somatização; Tentativa de Suicídio; Trauma do Estupro; Vínculo, Prejudicado; Violação de Direitos; Volição, Prejudicada.
Domínio dos outros	Adaptação Comunitária, Prejudicada; Capacidade da Família para Participar no Planejamento do Cuidado; Comunicação Familiar, Prejudicada; Conhecimento do Cuidador, Prejudicado; Convivência, Conflituosa; Corresponsabilização, Prejudicada; Crença da Família, Conflituosa; Crise Familiar; Desenvolvimento Comunitário, Prejudicado; Enfrentamento do Cuidador, Prejudicado; Imaginário Popular, Interrompido; Liderança Comunitária, Prejudicada; Papel Comunitário, Prejudicado; Papel Grupal, Prejudicado; Participação Comunitária, Prejudicada; Planejamento Familiar, Prejudicado; Risco de Conhecimento do Cuidador, Prejudicado; Risco de Desenvolvimento Comunitário, Prejudicado; Risco de Violência na Comunidade; Violência Doméstica; Violência na Comunidade.
Domínio do mundo	Acompanhamento Longitudinal, Prejudicado; Ambiente Domiciliar, Prejudicado; Barreiras Culturais; Clínica Ampliada, Ineficaz; Ecologia, Prejudicada; Perspectiva Intersetorial, Prejudicada; Reabilitação Psicossocial, Prejudicada.
**Conceitos do *Tidal Model***	**Resultados de enfermagem constantes na CIPE® 2019/2020**
Domínio do eu	Abuso de Drogas, Ausente (10028868); Aceitação da Condição de Saúde (10023499); Adesão ao Regime Medicamentoso (10030192); Adesão ao Regime Terapêutico (10030205); Agitação, Reduzida (10027843); Alergia, Ausente (10047492); Ansiedade, Reduzida (10027858); Apetite, Positivo (10040333); Autoestima, Positiva (10025751); Autoimagem, Positiva (10027108); Automutilação, Ausente (10029106); Capacidade para Proteção, Eficaz (10028276); Capaz de Executar a Manutenção da Saúde (10023452); Comportamento Sexual, Eficaz (10028187); Condição Espiritual, Eficaz (10028529); Desempenho de Papel, Eficaz (10027940); Desenvolvimento Humano, Eficaz (10028572); Desenvolvimento Infantil, Eficaz (10030222); Diarreia, Ausente (10040063); Efeito Colateral da Medicação, Ausente (10040295); Enfrentamento, Eficaz (10022378); Esperança (10025780); Estresse, Diminuído (10027929); Fadiga, Ausente (10034727); Hidratação, Adequada (10042065); Humor Deprimido, Diminuído (10027901); Identidade Pessoal, Positiva (10025664); Impotência, Diminuída (10027120); Ingestão de Alimentos, Melhorada (10047324); Ingestão de Líquidos, Melhorada (10047330); Interação Medicamentosa Adversa, Ausente (10042991); Memória, Eficaz (10028435); Náusea, Ausente (10028984); Negação, Ausente (10044260); Percepção Sensorial, Eficaz (10028173); Percepção, Melhorada (10047437); Resposta ao Trauma do Estupro, Melhorada (10027668); Resposta ao Trauma, Melhorada (10027760); Sono, Adequado (10024930); Tolerância à Atividade, Eficaz (10027634); Tomada de Decisão, Eficaz (10028731); Violência, Ausente (10029168); Vômito, Ausente (10029181).
Domínio dos outros	Apoio Familiar, Positivo (10045702); Cuidador Capaz de Executar o Cuidado (10035405); Enfrentamento Comunitário, Eficaz (10034801); Enfrentamento do Cuidador, Eficaz (10034838); Enfrentamento Familiar, Eficaz (10034770); Estresse do Cuidador, Reduzido (10027794); Parentalidade, Eficaz (10027955); Processo Familiar, Eficaz (10025232).
**Conceitos do *Tidal Model***	**Resultados de enfermagem não constantes na CIPE® 2019/2020**
Domínio do eu	Acatisia, Melhorada; Acidente, Ausente; Adaptação, Melhorada; Alteração Hematológica, Melhorada; Alucinação, Melhorada; Angústia, Diminuída; Aprendizagem, Melhorada; Ataxia, Melhorada; Atenção, Melhorada; Atividade Psicomotora, Melhorada; Autonomia, Eficaz; Autorrealização, Exitosa; Bruxismo, Melhorado; Bulimia, Ausente; Campo de Energia, Melhorado; Capacidade para Executar Atividades de Vida Diária (especificar), Exitosa; Capacidade para Manejar (Controlar) o Regime Medicamentoso, Exitosa; Capacidade para Participar no Planejamento do Cuidado, Exitosa; Capacidade para Participar, Exitosa; Capacidade para Proteção, Eficaz; Catatonia, Melhorada; Comportamento Agressivo, Melhorado; Comportamento Autodestrutivo, Melhorado; Comportamento Compulsivo, Melhorado; Comportamento Desorganizado, Melhorado; Comportamento Repetitivo, Melhorado; Comunicação Terapêutica, Eficaz; Comunicação, Eficaz; Concentração, Eficaz; Condição Social, Melhorada; Confiança, Melhorada; Confusão, Melhorada; Conhecimento sobre Educação Sexual, Melhorado; Conhecimento sobre Modelo Manicomial, Melhorado; Conhecimento sobre Regime Medicamentoso, Melhorado; Consciência de Si Mesmo, Aumentada; Constipação, Melhorada; Controle do Impulso, Eficaz; Controle do sintoma de abstinência, Eficaz; Convulsão, Ausente; Crença Cultural, Positiva; Crença de Saúde, Positiva; Crença Religiosa, Positiva; Crescimento, Eficaz; Criatividade, Fortalecida; Crise Acidental, Melhorada; Crise Maturacional (de Desenvolvimento), Melhorada; Crise Psicótica, Melhorada; Crise Situacional, Melhorada; Culpa, Diminuída; Delírio, Melhorado; Dentição, Melhorada; Dependência de Drogas, Melhorada; Desamparo, Melhorado; Desempenho Escolar, Eficaz; Desempenho Sexual, Melhorado; Desenvolvimento do Adolescente, Eficaz; Desinstitucionalização, Eficaz; Desintoxicação, Eficaz; Eliminação, Eficaz; Emancipação, Eficaz; Embotamento Afetivo, Melhorado; Emoção, Positiva; Empatia, Melhorada; Estado Maníaco, Melhorado; Estigma, Diminuído; Estratégia de Redução de Danos, Eficaz; Euforia, Melhorada; Exercício Físico, Melhorado; Expressão de Sentimento, Eficaz; Frustração, Melhorada; Hiperatividade, Melhorada; Hipertermia, Ausente; Hipertireoidismo, Ausente; Hipoatividade, Melhorada; Hipocondria, Melhorada; Hipomania, Melhorada; Hipotireoidismo, Ausente; Identidade de Gênero, Fortalecida; Inclusão, Eficaz; Infecção Sexualmente Transmissível, Ausente; Intoxicação Medicamentosa (Carbonato de Lítio), Ausente; Intoxicação Medicamentosa, Ausente; Linguagem, Melhorada; Mágoa, Melhorada; Medo, Diminuído; Metabolismo, Melhorado; Migração, Melhorada; Negligência, Ausente; Obsessão, Melhorada; Papel de Lazer, Eficaz; Pensamento, Melhorado; Pesadelo, Diminuído; Peso, Eficaz; Privacidade, Melhorada; Problematização, Melhorada; Processo Cardíaco, Eficaz; Processo de Luto, Melhorado; Processo do Sistema Gastrointestinal, Positivo; Prurido, Ausente; Pseudociese (Gravidez Psicológica), Melhorada; Psicose, Melhorada; Raiva, Diminuída; Reflexão, Melhorada; Regime Dietético, Melhorado; Relacionamento, Positivo; Revitimização, Ausente; Rotina, Melhorada; Satisfação Conjugal, Melhorada; Sinal Vital (Sinais Vitais), Melhorado; Socialização, Eficaz; Solidão, Diminuída; Somatização, Melhorada; Sonolência, Melhorada; Tentativa de Suicídio, Ausente; Trauma do Estupro, Ausente; Tristeza, Melhorada; Vergonha, Melhorada; Vínculo, Satisfatório; Violação de Direitos, Ausente; Violação de Direitos, Ausente; Volição, Melhorada.
Domínio dos outros	Adaptação Comunitária, Facilitada; Atitude Familiar, Melhorada; Capacidade da Família para Participar no Planejamento do Cuidado, Exitosa; Comunicação Familiar, Eficaz; Conhecimento do Cuidador, Fortalecido; Convivência, Melhorada; Corresponsabilização, Eficaz; Crença da Família, Positiva; Desenvolvimento Comunitário, Eficaz; Imaginário Popular, Fortalecido; Liderança Comunitária, Eficaz; Papel Comunitário, Eficaz; Papel Grupal, Eficaz; Participação Comunitária, Melhorada; Planejamento Familiar, Eficaz; Violência Doméstica, Ausente; Violência na Comunidade, Melhorada.
Domínio do mundo	Acompanhamento Longitudinal, Melhorado; Ambiente Domiciliar, Melhorado; Barreiras Culturais, Ausentes; Clínica Ampliada, Eficaz; Ecologia, Melhorada; Perspectiva Intersetorial, Eficaz; Reabilitação Psicossocial, Exitosa.

Os enunciados de DE/RE, com suas respectivas definições operacionais e enquadramentos com o *Tidal Model,* estão expostos na íntegra na seção de material suplementar.

## DISCUSSÃO

Os achados do presente estudo apresentam o quantitativo majoritário de 520 (58,4%) de termos/conceitos da CIPE®, provenientes do Modelo de Sete Eixos, em relação aos termos/conceitos identificados. O mesmo não aconteceu em relação aos enunciados da CIPE® pré-coordenados, pois somente 146 (34,3%) dos enunciados de DE/RE construídos tiveram alguma equivalência com os enunciados pré-coordenados da terminologia.

É importante destacar que o quantitativo reduzido de enunciados da CIPE® pré-coordenados não significa ausência na terminologia, tendo em vista a grande quantidade de termos do eixo foco que serviram como base para a construção dos enunciados de DE/RE inéditos. São exemplos: Abstinência (Afastamento ou Retirada de Algo) (10035422); Ansiedade da Separação (10017880); Angústia (10006118); Bulimia (10003759); Catatonia (10004056); Comportamento, Desorganizado (10006059); Concentração (10004910); Desempenho Escolar (10017559); Discriminação (10006037); Hiperatividade (10009302); Hipoatividade (10009466)^(1)^.

Diante da representação dentro do Modelo de Sete Eixos, ressalta-se que o quantitativo de 440 (71,4%) enunciados de DE/RE não constantes pode indicar que o contexto da especialidade de saúde mental comunitária possui especificidades importantes que demandam maior representação nos enunciados pré-coordenados da CIPE®^(6,7)^.

Entre essas especificidades do contexto da especialidade, é possível destacar a importância de criação dos seguintes DE/RE: Intoxicação Medicamentosa (Carbonato de Lítio); Risco de Intoxicação Medicamentosa (Carbonato de Lítio); Intoxicação Medicamentosa (Carbonato de Lítio), Ausente; Alteração Hematológica; e Alteração Hematológica, Melhorada. Esses enunciados estão relacionados a repercussões de algumas medicações psiquiátricas que afetam diretamente o processo de saúde-doença dos usuários, além de fazerem parte da rotina dos enfermeiros que atuam na área^(15)^.

Os enunciados de DE/RE, como Conhecimento sobre Regime Medicamentoso, Prejudicado, Risco de Conhecimento sobre Regime Medicamentoso, Prejudicado e Conhecimento sobre Regime Medicamentoso, Melhorado, reforçam as possibilidades no âmbito da psicoeducação, sendo outra dimensão rotineira da prática dos enfermeiros que atuam na saúde mental^(5,15)^.

A partir da construção dos enunciados de DE/RE, tais como Crise Acidental, Crise Maturacional (de Desenvolvimento), Crise Situacional, Crise Acidental, Melhorada, Crise Maturacional (de Desenvolvimento), Melhorada e Crise Situacional, Melhorada, evidenciou-se que estes são exemplos de fenômenos rotineiros que fazem parte do vocabulário do contexto da saúde mental, mas que ainda não tinham representação como enunciados de DE/RE na CIPE® 2019/2020^(15)^.

O conjunto de DE/RE relacionados ao conceito de domínio do eu teve maior quantitativo, pois representa termos/conceitos e achados clínicos que dizem respeito à dimensão pessoal, privada e individualizada do ser humano, que está em constante dinâmica de reconstrução e mudança^(9)^. O conjunto de DE/RE relacionados ao domínio dos outros se concentra em representar termos/conceitos que abordam dinâmicas interpessoais e sociais, abrangendo aspectos dos papéis sociais, familiares, profissionais, comunitários, entre outros^(5,8)^.

O domínio do mundo é um conceito sustentado na compreensão do mundo da experiência de forma ampla, permeando processos relativos ao domínio do eu e dos outros, sendo o meio de compartilhamento das experiências vividas por cada pessoa e coletividades de forma singular^(5,9)^.

O enquadramento dos enunciados de DE/RE em três conceitos do *Tidal Model* proporciona parcimônia e flexibilidade para um uso menos restritivo, principalmente ao considerar a multiplicidade de abordagens teóricas no campo da saúde mental. No que se refere aos enunciados de DE/RE construídos baseados nas fundamentações epistemológicas do *Tidal Model*, destacam-se: Autorrealização, Prejudicada; Autorrealização, Exitosa; Dificuldade na Autrotranscendência; Autotranscendência, Exitosa; Emancipação, Eficaz; Emancipação, Prejudicada^(1,10)^.

A construção de enunciados de DE/RE para o contexto da saúde mental possui relevância não só em relação aos achados clínicos presentes na especialidade, mas também em relação aos paradigmas existentes na política de saúde mental brasileira: na RAPS do Sistema Único de Saúde. Essas nuances podem ser percebidas a partir dos seguintes DE/RE: Desinstitucionalização, Prejudicada; Desinstitucionalização, Eficaz; Corresponsabilização, Prejudicada; Corresponsabilização, Eficaz; Reabilitação Psicossocial, Prejudicada; Reabilitação Psicossocial, Exitosa; entre outros^(5,7)^.

A RAPS é composta por um conjunto de níveis de atenção à saúde destinados ao atendimento de pessoas em sofrimento psíquico e em uso problemático de drogas. Compreende atenção básica, atenção psicossocial, atenção de urgência e emergência, atenção residencial de caráter transitório, atenção hospitalar, estratégias de desinstitucionalização e estratégias de reabilitação psicossocial. A construção dos enunciados de DE/RE buscou valorizar a prática dos diferentes serviços envolvidos na rede de atenção, inclusive as diferentes modalidades dos Centros de Atenção Psicossocial^(5,7)^.

Considerando a incorporação dos DE/RE da CIPE® nos sistemas de informação em saúde, destaca-se sua relevância em relação à implementação do PE na prática clínica, tendo em vista que são baseados em normas da ISO. Essas normas proporcionam padronização de enunciados de diagnósticos, resultados e ações de enfermagem segundo uma representação computacional da prática de enfermagem^(16)^.

Essa padronização favorece documentações eletrônicas recuperáveis e interoperáveis, além da padronização de termos/conceitos da prática de enfermagem. O uso de uma linguagem unificada promove melhora na comunicação entre os próprios profissionais e entre profissional-paciente, impactando positivamente a segurança do paciente, mitigando erros e riscos de danos desnecessários associados ao cuidado em saúde^(17)^.

Entende-se que os DE/RE da CIPE® também fornecem ganhos ao explorar diferentes conceitos e fenômenos da prática profissional, podendo se tornar uma base conceitual para a criação de teorias de enfermagem, como visto no estudo^(18)^ que descreve uma estratégia de teorização que integra componentes de classificações e terminologias com elementos de teorias de enfermagem de grande e médio alcance.

Diante do exposto, percebe-se que esses diferentes aspectos abordados em relação aos enunciados de DE/RE para saúde mental comunitária dialogam com o Relatório Mundial de Saúde Mental da OMS e com o seu Plano de Ação Integral em Saúde Mental (2013-2030), contemplando três objetivos gerais propostos, sendo eles: 1) fornecer cuidados de saúde mental e sociais abrangentes, integrados e responsivos em contextos comunitários; 2) implementar estratégias de promoção e prevenção em saúde mental; e 3) de fortalecer os sistemas de informação, evidência e pesquisa para a saúde mental^(19)^.

### Limitações do estudo

Tem-se como limitação a necessidade de alinhamento dos resultados com a nova ISO 18.104:2023, pois não tinha sido publicada a tempo da finalização deste estudo. Evidencia-se que o rigor do estudo possibilita alinhamento dos resultados com suprimento dessas limitações na estruturação futura do subconjunto terminológico da CIPE®. O processo de validação do conteúdo das definições operacionais será objeto de estudo de pesquisas futuras, tendo em vista sua importância para a segurança na prática clínica.

### Contribuições para a área da enfermagem

A construção de DE/RE da CIPE®, associados ao referencial teórico do *Tidal Model*, torna-se uma estratégia potencializadora do PE na saúde mental comunitária, podendo favorecer a singularidade do cuidado, o ordenamento dos processos de trabalho, a segurança do paciente e a melhoria da qualidade dos registros profissionais, além de facilitar a difusão do conhecimento na especialidade.

## CONCLUSÕES

Conforme se objetivou, realizou-se a construção 426 enunciados de DE/RE da CIPE® para o contexto da saúde mental comunitária, sendo categorizados utilizando a teoria do *Tidal Model*. Os enunciados de DE/RE relacionados ao domínio do eu tiveram maior quantitativo, seguidos pelo domínio do outro e domínio do mundo. Evidenciou-se que a maioria dos enunciados de DE/RE da CIPE® pré-coordenados não constava na terminologia da CIPE® 2019/2020. A continuidade da pesquisa se dará na validação de conteúdo dos DE/RE e na construção e validação de intervenções de enfermagem.

## Data Availability

Os dados de pesquisa estão disponíveis em repositório: https://doi.org/10.48331/scielodata.NHZIL6.
